# On the Usefulness of Two Small-Scale In Vitro Setups in the Evaluation of Luminal Precipitation of Lipophilic Weak Bases in Early Formulation Development

**DOI:** 10.3390/pharmaceutics12030272

**Published:** 2020-03-16

**Authors:** Patrick J. O’Dwyer, Georgios Imanidis, Karl J. Box, Christos Reppas

**Affiliations:** 1 Pion Inc. (UK) Ltd., Forest Row, East Sussex RH18 5DW, UK; patrick.odwyer@ucc.ie (P.J.O.); kbox@pion-inc.com (K.J.B.); 2 Department of Pharmacy, School of Health Sciences, National and Kapodistrian University of Athens, GR 157 84 Zografou, Greece; 3 School of Life Sciences, Institute of Pharma Technology, University of Applied Sciences Northwestern Switzerland, Hofackerstrasse 30, 4132 Muttenz, Switzerland; georgios.imanidis@fhnw.ch; 4 Department of Pharmaceutical Sciences, University of Basel, CH 4056 Basel, Switzerland

**Keywords:** precipitation, supersaturation, biorelevant, in vitro techniques, PBPK modelling, biphasic dissolution, dissolution-permeation

## Abstract

A small-scale biphasic dissolution setup and a small-scale dissolution-permeation (D-P) setup were evaluated for their usefulness in simulating the luminal precipitation of three lipophilic weak bases—dipyridamole, ketoconazole and itraconazole. The transition from the gastric to intestinal environment was incorporated into both experimental procedures. Emulsification during the biphasic dissolution experiments had a minimal impact on the data, when appropriate risk mitigation steps were incorporated. Precipitation parameters estimated from the in vitro data were inputted into the Simcyp^®^ physiologically based pharmacokinetic (PBPK) modelling software and simulated human plasma profiles were compared with previously published pharmacokinetic data. Average C_max_ and AUC values estimated using experimentally derived precipitation parameters from the biphasic experiments deviated from corresponding published actual values less than values estimated using the default simulator parameters for precipitation. The slow rate of transport through the biomimetic membrane in the D-P setup limited its usefulness in forecasting the rates of in vivo precipitation used in the modelling of average plasma profiles.

## 1. Introduction

Weakly basic drugs usually have no solubility issues in the acidic environment of the stomach. However, due to the increased pH in the small intestine, concentrations (especially of lipophilic weak bases) arriving in the small intestine could supersaturate and/or precipitate in the contents of the small intestine and accordingly impact (at least early) drug exposure. Many in vitro methodologies have been proposed in an attempt to better evaluate this dynamic in vivo dissolution process [1].

One limitation of many relevant methodologies, especially of the so-called small-scale in vitro methodologies which are used early in the drug development process when available amounts of the new active pharmaceutical ingredient (API) are limited, is their inadequate validation with human data. Another issue with many of the in vitro methodologies is the lack of simulation of drug disappearance from bulk luminal contents, due to the absorption process. As a result, in many situations, overestimation of precipitation has been reported [1].

The use of an organic layer, such as octanol or decanol, on top of an aqueous layer can incorporate an absorption step into the dissolution experiment. The organic layer acts as a quasi-sink and unionised drug can partition from the aqueous layer into the organic layer, thus mimicking the absorption step in the small intestine. This allows the dynamic dissolution process, incorporating dissolution, supersaturation, absorption and precipitation to be observed simultaneously. However, the suitability and biorelevance of the biphasic dissolution methodology has been questioned. As the organic layer is in direct contact with the aqueous media, this can produce effects which are quite distinct from the absorption process in the human intestine. Some of the organic layer could become solubilised in the aqueous media and an emulsification could be formed as a result. This is especially an issue when surfactants are present in the media, such as in Level II biorelevant media containing bile salts and phospholipids [2], or in the formulation, which is typical for bio-enabling formulations. Many groups have studied biphasic dissolution experiments as a method to improve the predictive capabilities compared to single phase dissolution [3,4,5,6,7,8,9,10,11,12]. The majority of these experiments have been carried out using full scale dissolution apparatus. The inForm (Pion Inc., Billerica, MA, USA) is a small-scale automated dissolution platform which employs in situ fibre optic UV dip probes to measure drug concentration in real time. In addition, it uses a potentiometric pH probe to monitor pH of the media in real time to facilitate in situ pH control. Real-time analytics avoids errors of sample aging, increases sampling frequency and requires fewer manipulations to be carried out during the experiment [13]. A small-scale biphasic dissolution test has been developed using the inForm containing an aqueous layer (*V* = 40 mL) and an organic lipophilic layer (Decanol *V* = 40 mL) [14].

Another small-scale in vitro method for evaluating absorption of drug in the small intestine is by using an artificial membrane. The μFLUX (Pion Inc., Billerica, MA, USA) is a small-scale dissolution—permeation (D-P) system consisting of four pairs of two chambers setups separated by an artificial biomimetic membrane [15,16,17]. As the system has four pairs of chambers, experiments can be run in parallel, facilitating a high experimental throughput. Drug is quantified in situ in all chambers by eight UV fibre optic dip probes connected to the Rainbow instrument (Pion Inc.). Calculation of in vivo precipitation rates was not the intended primary purpose of the µFLUX setup and, therefore, it has not been optimised for this application. It has previously been shown to have other applications during drug development, such as examining the differences in bioavailability of different formulations [15,16] or assessing the effects of a raised gastric pH [17].

The aim of this study was to assess the performance of three lipophilic weak bases (dipyridamole, ketoconazole and itraconazole) using a small-scale biphasic dissolution setup (based on the inForm platform) and a small-scale D-P apparatus (the µFLUX apparatus), and to evaluate the usefulness of the collected precipitation data in simulating the plasma profile after administration in the fasted state. As intraluminal in vivo precipitation data are scarcely available, plasma data were used in this evaluation, providing a comprehensive view on the usefulness of the approach. Both in vitro setups included the transition from an acidic gastric environment to the almost neutral environment of the intestine. For all three drugs, the precipitation parameters from the in vitro tests were incorporated into physiologically based pharmacokinetic (PBPK) models and the simulated plasma profile was evaluated vs. previously published pharmacokinetic data in adults.

## 2. Materials and Methods

### 2.1. Materials

Dipyridamole powder was obtained from Sigma-Aldrich (Steinheim, Germany). Ketoconazole was obtained from Fisher Scientific (Leics, UK). Itraconazole powder was kindly donated from Shin-Etsu (Stevenage, UK).

Dipyridamole formulation was an in-house prepared 10 mg/mL aqueous suspension containing 0.5% *w*/*v* methylcellulose. The suspension was prepared every experimental day. The ketoconazole dose consisted of the unformulated active ingredient. Sporanox^®^ 10 mg/mL oral solution (OS) and 100 mg capsules (Janssen–Cilag Ltd, Bucks, UK) were sourced from a local community pharmacy. Sporanox^®^ OS uses hydroxypropyl-β-cyclodextrin which acts as a solubiliser of itraconazole, whereas Sporanox^®^ capsules consist of an amorphous dispersion of itraconazole and hypromellose on the surface of inert sugar cores.

Fluorene was received from Sigma-Aldrich. Methylcellulose was received from Shin-Etsu. Acceptor Sink Buffer (ASB), consisting of a HEPES buffer at pH 7.4 along with surfactants, and GIT (Gastro Intestinal Tract) Lipid Solution (20% lecithin in dodecane lipid solution) were received from Pion Inc. FaSSIF V2 powder was obtained from biorelevant.com (London, UK), which results in a final sodium taurocholate and lecithin concentration of 3 and 0.2 mM, respectively, when reconstituted to its final intestinal concentration [2]. Further information on the preparation of FaSSIF V2 for use in these experimental setups can be found in Section 2.2.2 and Section 2.2.3. Decanol was purchased from Alfa Aesar (Heysham, UK). Hard gelatin capsules were purchased from Agar Scientific Ltd (Essex, UK). All other chemicals and solvents were of analytical grade or HPLC grade and purchased from Fisher Scientific UK.

### 2.2. Methods

#### 2.2.1. Physicochemical and Pharmacokinetic Properties of Model Drugs

Log P and pK_a_ values for all APIs were taken from previously experimentally measured in-house values measured using the Sirius GLpKa, Sirius T3 (Pion Inc.) or inForm platforms. As the solubility of itraconazole at intestinal pH was below the LOQ in these experiments, a literature value was taken for the solubility of itraconazole in Level II biorelevant intestinal media [18]. Equilibrium solubility of dipyridamole and ketoconazole in 0.1 M HCl was measured with the shake-flask method. Briefly, an excess of API was weighed out and media added. The samples were shaken at 1200 rpm for 24 h at 37 °C using the Ventura 2000 (Mikura, West Sussex, UK). The samples were filtered through 0.1 μm filters (Minisart^®^ High Flow Syringe Filter 16553K polyethersulfone) and concentration was measured by HPLC. The HPLC analysis was carried out using a Waters ACQUITY Arc (Waters Corporation, MA, USA) equipped with a detector (2998 PDA Detector, auto sampler (Sample Manager FTN-R), solvent manager (Quaternary Solvent Manager-R) and Waters CORTECS C18 (2.7 um 4.6 × 50 mm) column. The mobile phase comprised a mixture of acetonitrile and 0.1% formic acid (*v*/*v*) using a gradient flow rate. The detection wavelengths for dipyridamole and ketoconazole were 281 and 291 nm, respectively. A linear relationship was established between drug concentration and area under the peak for both dipyridamole (*R*^2^ = 0.9997) and ketoconazole (*R*^2^ > 0.9999) in the ranges of 0.5–100 µg/mL and 1–100 µg/mL respectively. The remaining solubility experiments for dipyridamole, ketoconazole and itraconazole in Level II FaSSIF V2, 0.1 M HCl (itraconazole only), pH 7.4 buffer and in decanol were carried out using the inForm or μDISS Profiler™ (Pion Inc.) platforms. An excess of API was weighed out into each vessel and media was added. This was stirred at 300 rpm for 24 h at 37 °C and drug was quantified using multi-wavelength fibre optic dip probes. Different standard spectra were collected for the neutral and ionised forms of each compound and a linear relationship (*R*^2^ > 0.99) was established between absorbance and concentration in each case, with the detection wavelengths shown in the supplementary information Table S1. All solubility experiments were performed in triplicate.

Pharmacokinetic absorption and distribution properties were estimated using the physiochemical properties and in vitro experiments as outlined in Table 1, Table 2 and Table 3 for dipyridamole, ketoconazole and itraconazole, respectively. Elimination characteristics of the model drugs were estimated from previously published data after intravenous administration of dipyridamole [19], ketoconazole [20] and itraconazole [21].

#### 2.2.2. Preparation of Level II FaSSIF V2 × 10 Concentrated for Biphasic Dissolution Experiments

FaSSIF V2 was prepared using a 0.01 M acetate phosphate buffer at a pH of 6.8. The acetate phosphate buffer was selected as it is the primary buffer for the in situ pH control on the inForm platform. FaSSIF V2 powder (0.9 g) was added to approx. 25 mL of buffer. This was stirred until all the powder had dissolved and then made to volume (50 mL) with buffer. This was left for two hours at room temperature before use.

#### 2.2.3. Preparation of Level II FaSSIF V2 × 4 Concentrated for D-P Experiments

FaSSIF V2 was prepared by using a 0.113 M phosphate buffer. The phosphate buffer was selected due to the strong UV interference of the standard FaSSIF V2 maleate buffer. The pH of the phosphate buffer was adjusted to ensure a final pH of 6.8 after addition of the concentrated intestinal media to the gastric media. FaSSIF V2 powder (0.36 g) was added to approx. 25 mL of buffer. This was stirred until all the powder had dissolved and then made to volume (50 mL) with buffer. This was left for two hours at room temperature before use.

#### 2.2.4. Dose Selection

The target dosing strategy was to scale down to the clinical dose administered with a glass of water (i.e., 75, 200, and 200 mg for dipyridamole, ketoconazole, and itraconazole, respectively, with 250 mL water) [22,23,24]. For the biphasic experiments, since 40 mL of aqueous medium was used, the dipyridamole, ketoconazole and itraconazole doses were estimated to be 12, 32, and 32 mg, respectively. However, in the biphasic experiments, as the drug was quantified in real time by the in situ UV dip probes, it was not possible to dilute the sample before quantification. In order to remain in the linear range of the UV-Vis absorbance, a dose of 10 mg was finally selected for dipyridamole experiments. For ketoconazole and itraconazole, the selected doses were 20 and 5 mg, respectively, based on their solubilities in decanol and the aim for the decanol layer to act as an absorption sink. The volume of decanol (40 mL) used in the biphasic experiment was over 6 times the minimum volume needed to dissolve the 10 mg dose of dipyridamole, 4.85 times the minimum volume to dissolve the 20 mg ketoconazole dose and 1.79 times the minimum volume required to dissolve the itraconazole dose. A balance was sought between achieving sink conditions and using therapeutically relevant doses. For the D-P experiments, the dose was further scaled down by a factor of two to account for the difference in the simulated intestinal volumes between the two setups (20 mL for the D-P setup).

#### 2.2.5. Biphasic Dissolution Test

**Figure 1:**
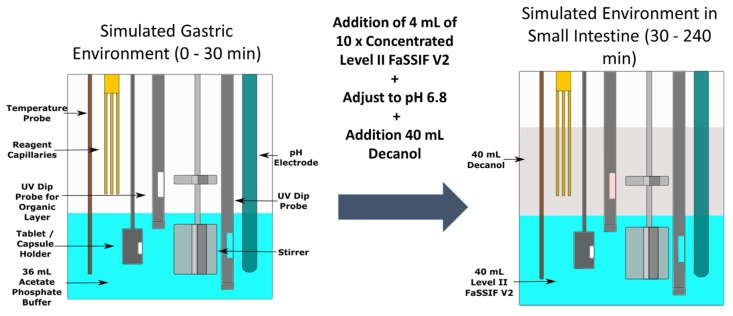
Schematic of the biphasic dissolution test using the inForm platform.

The biphasic experiment setup is outlined in Figure 1. For solid dosage forms (Sporanox^®^ capsules and unformulated ketoconazole API), the dose was introduced into a new hard gelatin capsule (volume 0.37 mL, diameter 6.0 mm), which was delivered into the aqueous layer using an automated sample handling mechanism. For all other formulations (Sporanox^®^ OS and dipyridamole suspension), the dose was introduced into the aqueous compartment using an automatic liquid handling needle. The dose was introduced into 36 mL of a 0.01 M acetate phosphate buffer at pH 2 representing the gastric environment in a cylindrical vessel (diameter 50.0 mm, height 74.9 mm). Thirty minutes after initiation of an experiment, 4 mL of 10 × concentrated Level II FaSSIF V2 and decanol (40 mL) were added, with the pH of the aqueous media increased to pH 6.8 using 0.5 M NaOH to simulate the transition into the upper small intestine. Stirring was temporarily suspended while the decanol was being added to avoid excessive turbulence. The concentration in both layers was determined every 120 s by two in situ multi-wavelength fibre optic UV probes, with the relevant detection wavelength ranges outlined in the supplementary material Table S1. The duration of experiment in the intestinal environment was 210 min. The pH was monitored throughout using the in situ pH probe and was maintained to ± 0.1 pH unit of the target pH, using 0.5 M HCl or 0.5 M NaOH. All experiments were carried out in triplicate at 37 °C. The stirring speed of the paddles in the vessel was set at 100 rpm.

#### 2.2.6. Biphasic Emulsification Risk Investigation

To assess the potential partitioning of surfactants from the aqueous to the organic layer, changes in surface tension of the aqueous layer were measured during the biphasic experiment. The biphasic experimental method was the same as outlined previously without adding any drug. Samples from the aqueous layer were taken every 30 min using the automated liquid handling needle and collected in a 96-well plate. Surface tension was measured using a Delta-8 tensiometer (Kibron Inc., Helsinki, Finland), an eight channel microtensiometer.

To investigate the risk of incorporation of decanol into the aqueous layer, fluorene was pre-dissolved as a lipophilic marker into the decanol at a concentration of 55 µg/mL. The biphasic experimental method was the same as outlined above. Changes in the fluorene concentrations were detected by the two fibre optic UV dip probes.

To investigate the risk of emulsification of the decanol into Level II FaSSIF V2, Level II FaSSIF V2 was saturated with decanol and equilibrium solubility experiments were carried out. FaSSIF V2 was initially prepared using a 0.01 M acetate phosphate buffer and adjusted to pH 6.8. An equal volume of decanol was added to the container and it was shaken for 60 s. It was then left to stand for 120 min. The aqueous layer was then extracted; this was considered to be “saturated” with decanol. Equilibrium solubility experiments were carried out, using the same method as outlined previously. The effect of the “saturated” FaSSIF on the equilibrium solubility of dipyridamole and ketoconazole was tested.

#### 2.2.7. D-P Experiments

**Figure 2:**
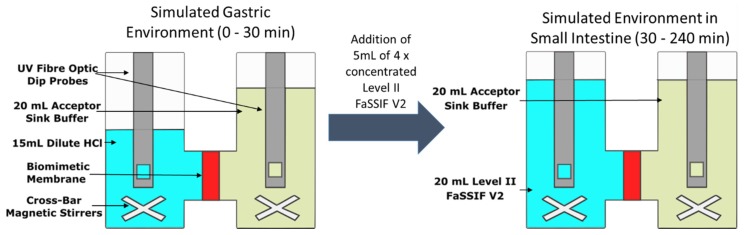
Schematic of the dissolution-permeation (D-P) test using the µFLUX apparatus.

The D-P setup is outlined in Figure 2. The two chambers were separated by a biomimetic membrane which consists of a 0.45 μm polyvinylidenfluoride membrane coated with 25 μL of the GIT lipid solution. The area of the membrane was 1.54 cm^2^. Stirring was provided by cross-bar magnetic stirrers in both chambers and was set at 150 rpm throughout the experiment. The acceptor chamber was filled with 20 mL of acceptor sink buffer (ASB) throughout the experiment, to maintain sink conditions during the experiment [15]. The donor chamber was initially filled with 15 mL of dilute HCl at pH 2 and drug was manually introduced to begin the experiment. Thirty minutes after the initiation of an experiment, 5 mL of 4 × concentrated solution of Level II FaSSIF V2 was added to the donor chamber. The pH of the 4 × concentrated Level II FaSSIF V2 solution was adjusted in order to achieve a final pH of 6.8 in the donor chamber. The temperature was controlled to 37 °C throughout the experiment. Drug was quantified using multi-wavelength UV dip probes, with probes inserted into both the donor and acceptor chambers.

#### 2.2.8. PBPK Modelling

PBPK modelling was carried out using the ADAM (Advanced Dissolution, Absorption, and Metabolism) model, which is available as part of the Simcyp PBPK simulator (Version 18, Release 2). Modelling was carried out in a stepwise fashion using an in vitro–in vivo extrapolation (*IVIV*_E) of dissolution and solubility, as outlined previously by Pathak et al. [25,30] and Hens et al. [35] Aqueous and bile micelle-mediated solubility data were estimated using the SIVA (Simcyp In Vitro Analysis) toolkit, using solubility data from Section 2.2.1 and other literature sources [18,25,30]. Initially, the intrinsic solubility and solubility factor were calculated using the experimental pH solubility profile in SIVA. The solubility factor is the ratio of the maximum aqueous solubility of the ionised form of the drug to the intrinsic solubility. The unionised (log Km:w neutral) and ionised (log Km:w ion) micellar: buffer partition coefficients were estimated using the biorelevant media solubility values in SIVA. Solubility values obtained from literature sources were used as external validation when solubility parameters had been established. Physicochemical and pharmacokinetic properties of the model drugs were estimated as described in Section 2.2.1. The diffusion layer model (DLM) scalar was used as part of the dissolution model in the simulator, with the value estimated from the dissolution profiles in SIVA in cases where models were sensitive to changes in the DLM value. The Kp scalar was used to adjust the predicted volume of steady state to improve the model to better reflect the in vivo values. It varies between molecules depending on how well the predicted volume of steady state fits in vivo data, with 1 being the default value (i.e., no scaling). The Kp scalar can be calculated based on animal or human data. In each case, the modelled profiles showed no sensitivity to the fraction unbound in enterocytes, so differences did not have any impact on the simulated profile.

The critical supersaturation concentration (CSC) was determined as the concentration at which precipitation begins. CSC could not be directly determined from the biphasic or D-P setup. To determine the CSC, a concentrated stock solution of drug was prepared using DMSO. Aliquots of stock solution were injected into the Level II FaSSIF V2 at pH 6.8 with stirring of 100 rpm at 37 °C using the inForm platform. Level II FaSSIF V2 was used to determine the CSC to match the composition of the aqueous intestinal media in the setups. Precipitation was detected by scattering in the UV/Vis spectrum at 600–700 nm, as none of the studied APIs have UV activity in this range. The concentration at which the precipitation initially occurs was determined to be the CSC. The critical supersaturation ratio (CSR) was calculated by dividing the CSC by the equilibrium solubility in Level II FaSSIF V2.

An empirical first-order precipitation rate constant (PRC) was employed for estimating precipitation in the simulator. The PRC was estimated by fitting aqueous concentration profiles using Microsoft Excel tools (Rich, WA) for dipyridamole and ketoconazole. Precipitation was considered to have terminated when the drug concentration in the aqueous layer reached equilibrium solubility. An example of this is shown in Supplementary Figure S1 for ketoconazole.

In the case of the itraconazole formulations, aqueous data was absent after transition to intestinal conditions due to concurrent large scattering and weak UV absorbance. Therefore, for the biphasic experiments, precipitation was estimated based on the changing partition rate into the decanol layer. The rate of partitioning into the decanol layer decreased rapidly before plateauing when the drug reaches equilibrium solubility in the aqueous phase. The time taken to reach this plateau rate provided an estimate of the time to reach equilibrium solubility. This was determined as the first time point at which the partition rate fell below double the equilibrium partition rate of the compound into the decanol layer, where the equilibrium rate had been calculated from a time range far beyond the termination of clinically relevant precipitation (over three hours after the transition to intestinal conditions in the biphasic experiments). Using this estimated time, a first order PRC was then calculated using Excel. To validate this workflow, the same process was carried out using ketoconazole and the results were compared to the profiles calculated from the measured aqueous concentration data. Ketoconazole was chosen to validate this workflow as it showed incomplete dissolution, similar to the itraconazole formulations. This process for ketoconazole is shown in Supplementary Figure S2. Due to the lag time associated with the flux of drug across the biomimetic membrane, this was not considered to be a reliable real-time indicator of precipitation occurring in the donor chamber, so it was not possible to adapt this workflow for the D-P experiments.

As the Sporanox OS and capsules employed different bio-enabling technology to enhance oral bioavailability, as mentioned in Section 2.1, this required additional parameters to be considered in the models. For the cyclodextrin formulations, the increase in bioavailability is accounted by using an excipient mediated solubility feature, using previously published binding itraconazole-cyclodextrin constants [33]. The equation to model this cyclodextrin-mediated solubility (1:2 binding model) is as follows:

$$S_{bound,excip}=S_{0}\times \left(K_{1:1}{\left[CD\right]}_{free}+K_{1:1}K_{1:2}{\left[CD\right]}_{free}^{2}\right)$$

where *K*_1:1_ and *K*_1:2_ are the binding constants associated with drug–cyclodextrin complexes, and *S*_0_ is the intrinsic solubility.

For the amorphous solid dispersion (ASD) present in the capsules, the model accounts for the increase in bioavailability by modelling two separate states. Amorphous solubility data [16,32] were used to account for the boost in solubility of the amorphous form of the drug. The precipitated drug in the model was converted back to the crystalline form of the drug. To further account for the differences between the bio-enabling formulations, separate PRCs were calculated for the OS and capsules. These separate PRCs will help to account for any inhibition of drug precipitation caused by the presence of cyclodextrins in the OS and hypromellose in the ASD.

To evaluate the usefulness of PRC values estimated from the biphasic and D-P experiments, PRC values were inputted into PBPK models. The models were used to simulate the plasma profile, and PK parameters from these simulated profiles were compared with average values from clinical studies in healthy adults, after single dose administration of immediate release tablet formulations of dipyridamole (75 mg) [22] and ketoconazole (200 mg) [23] and of Sporanox^®^ oral solution (200 mg) and Sporanox^®^ capsules (200 mg) [24]. Ten virtual trials using the same number of subjects as the respective clinical studies were simulated in each case and were conducted using the Sim-Healthy Volunteers population in the Simcyp software (version 18 release 2).

In addition, the PK parameters estimated from the plasma profiles simulated using the PRC and CSR values from the in vitro experiments performed in the present study were compared with those estimated from the plasma profiles simulated using the default values for PRC and CSR in Simcyp software (4 h^−1^ and 10, respectively) and using models in which no in vivo precipitation was simulated.

## 3. Results and Discussion

### 3.1. Data on the Emulsification Risk in the Biphasic Experiments

**Figure 3:**
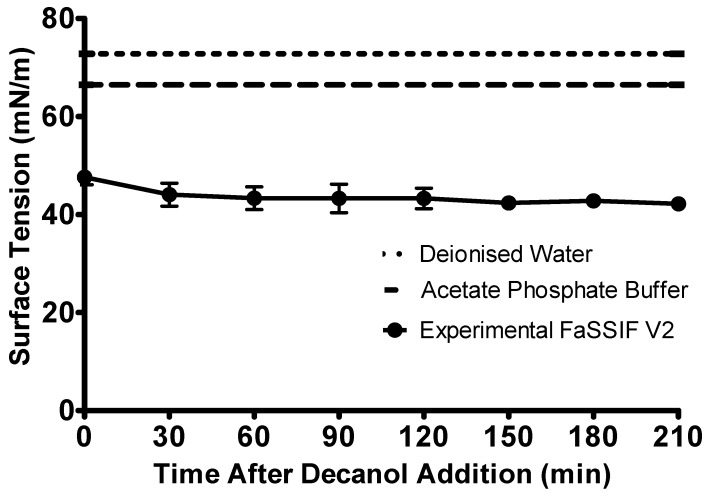
Change in the surface tension of the biorelevant media over the duration of the intestinal sector of the biphasic dissolution experiment. Surface tension of the FaSSIF V2 is represented by the circles, the surface tension of the acetate phosphate buffer is represented by the dashed line, the surface tension of deionised water is represented by the dotted line. Each data point represents the mean ± SD (*n* = 3).

The surface tension of the aqueous layer showed only a minimal difference throughout the biphasic experiment (Figure 3). The surface tension of the FaSSIF V2 remained significantly below the surface tension of the acetate phosphate buffer (66.50 ± 0.65 mN/m, *n* = 3, ± SD) for the whole experiment. The surface tension changed from 47.61 ± 1.51 mN/m (*n* = 3, ± SD) prior to the addition of the decanol layer to 42.18 ± 1.34 mN/m (*n* = 3, ± SD) at the end of the experiment. The minimal decrease in the surface tension observed throughout the experiment may be caused by the introduction of the sampling needle into the vessel every 30 min. As the needle must pass through the decanol layer to sample from the aqueous phase, it was likely that this sampling procedure could introduce some contamination into the aqueous layer. However, this sampling procedure was not part of the standard biphasic experimental procedure.

In the experiment investigating the risk of incorporation of decanol into the aqueous layer, the concentration of the lipophilic dye fluorene in the organic layer decreased from 56.72 ± 0.75 µg/mL (*n* = 3, ± SD) at the beginning of the experiment to 54.08 ± 1.33 µg/mL (*n* = 3, ± SD) three hours after decanol was added. Concentrations of fluorene in the aqueous layer were below the limit of quantification throughout the experiment. The small decrease in concentration of fluorene in decanol was thought to be due to the partitioning of fluorene into the FaSSIF V2. However, as the decrease in fluorene concentration in the decanol was <3 µg/mL and the concentration in the aqueous layer was below the limit of quantification, this was believed to be as a result of equilibrium being established between the two layers, as opposed to mixing of the decanol layer into the aqueous media.

When assessing the effect of saturating the biorelevant media with decanol, the equilibrium solubility of dipyridamole (11.99 ± 1.12 μg/mL for Level II FaSSIF V2 vs. 12.17 ± 0.78 μg/mL for decanol saturated Level II FaSSIF V2, *n* = 3, ± SD) and ketoconazole (15.92 ± 0.52 μg/mL for Level II FaSSIF V2 vs. 16.19 ± 0.51 μg/mL for decanol saturated Level II FaSSIF V2, *n* = 3, ± SD) were not found to be significantly different in the Level II FaSSIF V2 which had been “saturated” with decanol compared with the non-saturated Level II FaSSIF V2. Itraconazole was not selected to be tested using this setup as its solubility was below the limit of quantification, potentially resulting in erroneous results.

While directly quantifying changes in surfactant concentrations in the aqueous layer would be the optimum approach to assess any partitioning of surfactants, this was not possible using the analytical equipment available for this study. However, these series of experiments give confidence that the potential mixing of the organic layer with Level II FaSSIF V2 components had a minimal effect, at least when the stirring rate was not higher than 100 rpm.

### 3.2. Data from the Biphasic Experiments

**Figure 4:**
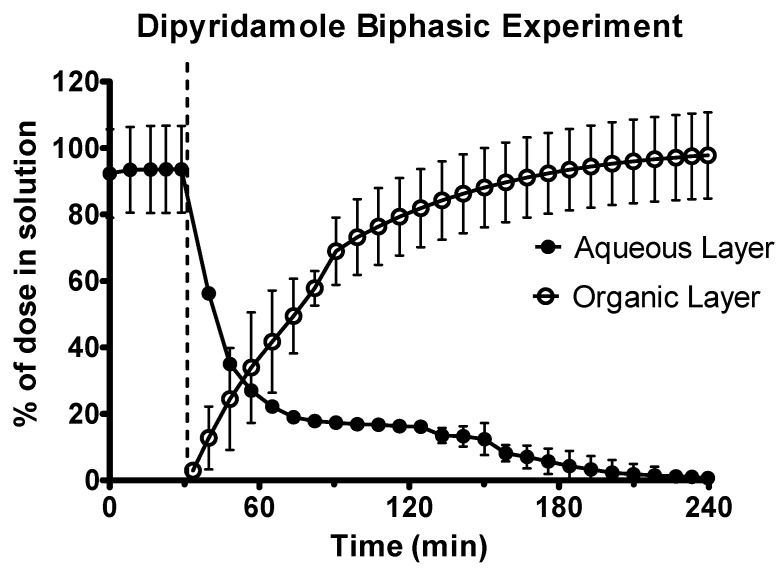
Drug release time profile for the dipyridamole oral suspension, dose equivalent to 10 mg active pharmaceutical ingredient (API). Aqueous layer release is represented by filled circles. Organic layer data are represented by the hollowed circles. Dotted line indicates transition from gastric sector to intestinal sector. Each data point represents the mean ± SD (*n* = 3).

The dipyridamole suspension was rapidly dissolved in the gastric sector, as shown in Figure 4. Upon transition to the intestinal environment, dipyridamole rapidly partitioned into the decanol layer. Precipitation of the dipyridamole was observed in the aqueous layer upon transition to intestinal pH 6.8, as observed by scattering in the UV spectrum. To calculate the concentration in the aqueous layer, this scattering was corrected using the Tyndall/Raleigh method [36] as part of the inForm refine software (version 1.6.11) (Pion Inc.). Precipitation had a minor effect for dipyridamole, as the total dose (>97%) of dipyridamole was present in the decanol layer at the end of the experiment, with precipitated drug redissolved as sink conditions were regenerated due to the rapid partitioning of drug into the decanol layer.

**Figure 5:**
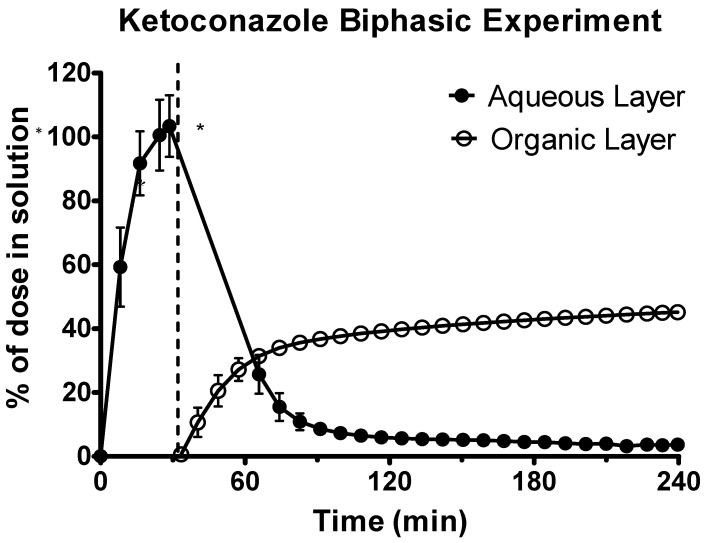
Drug release time profile for ketoconazole, dose equivalent to 20 mg API. Aqueous layer release is represented by filled circles. Organic layer data are represented by the hollowed circles. Dotted line indicates transition from gastric sector to intestinal sector. Each data point represents the mean ± SD (*n* = 3). * indicates where UV blackout has occurred in the aqueous phase due to precipitation.

Ketoconazole underwent rapid dissolution in the acidic gastric environment, as shown in Figure 5. Upon transition to the intestinal environment, there was significant turbidity in the aqueous phase causing some UV blackout. Therefore, the second derivative of the spectra was used to quantify drug in the aqueous environment. In the intestinal sector, ketoconazole initially rapidly partitioned into the decanol layer before the rate of partitioning plateaued. This plateau occurred with approx. 40% of the ketoconazole dose present in the decanol layer. This indicated that a significant quantity of drug had precipitated and was present in its solid state in the aqueous layer.

**Figure 6:**
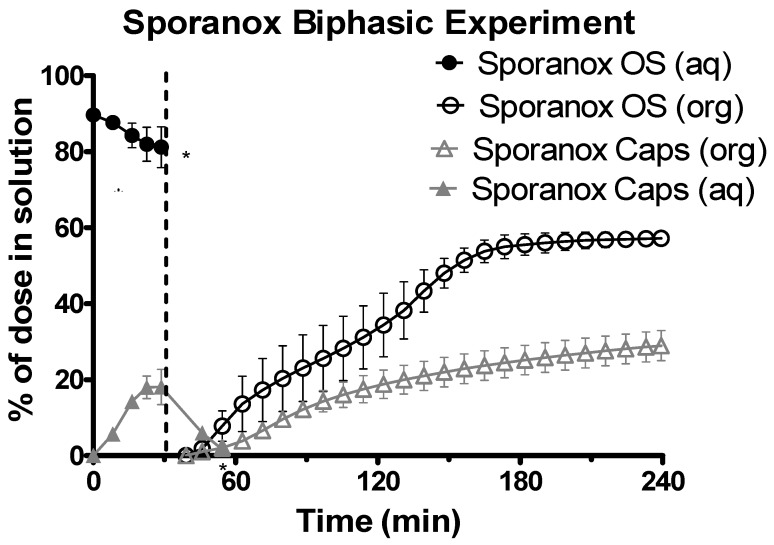
Drug release time profile for Sporanox^®^ formulations, dose equivalent to 5 mg API. Sporanox^®^ OS and Sporanox^®^ Capsules are represented by black circles and grey triangles respectively. Aqueous layer release is represented by filled points. Organic layer data are represented by the hollowed points. Dotted line indicates transition from gastric sector to intestinal sector. Each data point represents the mean ± SD (*n* = 3). * indicates where UV blackout occurred in the aqueous phase due to precipitation and/or limitations of quantification. aq = aqueous layer. org = organic layer.

The data from the aqueous phase for both of the Sporanox^®^ formulations was only available for the first hour (at most), as a combination of significant turbidity and low concentration resulted in a UV black out upon transition to the intestinal sector at pH 6.8 (Figure 6). At the end of the experiments in the gastric environment, there was a significantly greater concentration of itraconazole for the OS compared to the capsules (104.16 ± 6.88 μg/mL vs. 21.83 ± 1.60 μg/mL, *n* = 3, ± SD). Itraconazole concentrations in the organic phase at the end of the experiment were significantly higher (two tailed *t*-test *P* < 0.05) for the Sporanox^®^ OS compared to the Sporanox^®^ Capsules (Figure 6) (70.61 ± 2.01 μg/mL vs. 36.60 ± 5.67 μg/mL, *n* = 3, ± SD).

To further optimise the biphasic dissolution experiments, the different parameters of the biphasic dissolution experiment (vessel size, aqueous volume, organic volume and dose) could be scaled using a mathematical model relative to the first-order absorption rate constant of a particular drug to improve the physiological relevance of the setup, as outlined by Mudie et al. [37]. This level of optimisation was beyond the scope of this study but could improve the biorelevance of the biphasic dissolution setup employed in this study.

### 3.3. Data from the D-P Experiments

**Figure 7:**
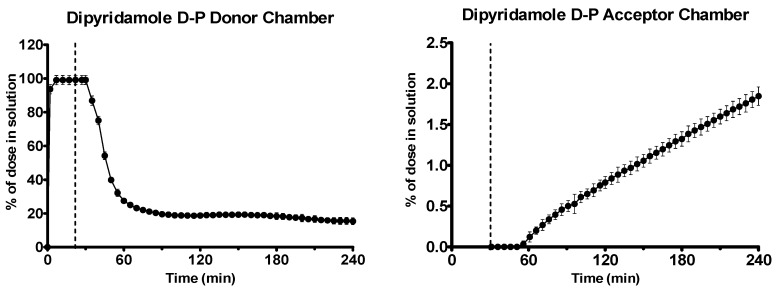
Drug release time profile for the dipyridamole dose equivalent to 5 mg API using the D-P apparatus. Left: Percentage release profile in the donor chamber. Right: Percentage release profile in the acceptor chamber. Dotted line indicates transition from gastric sector to intestinal sector. Each data point represents the mean ± SD (*n* = 3).

Dipyridamole was rapidly dissolved in the gastric environment, as shown in Figure 7. Upon transition to intestinal conditions, substantial precipitation occurred in the donor chamber, as shown by scattering in the UV spectrum. To quantify the concentration in the donor chamber, a scattering correction was applied using the AUPRO™ software (Pion Inc.). After an initial lag period, dipyridamole partitioned across the membrane into the acceptor chamber at a rate of 0.31 ± 0.02 μg·min^−1^·cm^−2^ (*n* = 3, ± SD).

**Figure 8:**
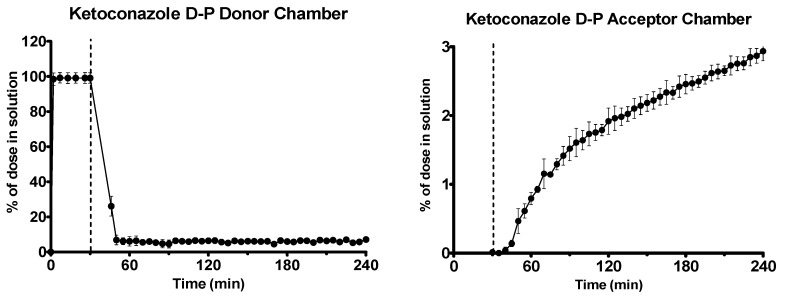
Drug release time profile for the ketoconazole dose equivalent to 10 mg API using the D-P apparatus. Left: Percentage release profile in the donor chamber. Right: Percentage release profile in the acceptor chamber. Dotted line indicates transition from gastric sector to intestinal sector. Each data point represents the mean ± SD (*n* = 3).

Ketoconazole dissolved rapidly in the gastric environment, as shown in Figure 8. After the shift to intestinal conditions, ketoconazole precipitated massively, as observed by scattering in the UV spectrum. The second derivative of the UV spectrum was used to quantify the drug due to the turbidity present. Early and late flux values of ketoconazole into the acceptor chamber were 2.06 ± 0.41 and 0.54 ± 0.04 μg·min^−1^·cm^−2^ (*n* = 3, ± SD), respectively.

**Figure 9:**
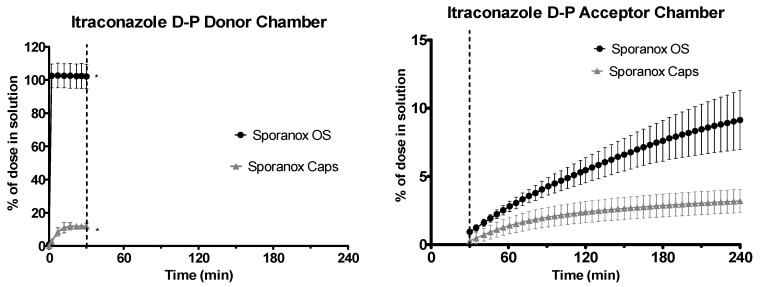
Drug release time profile for the itraconazole dose equivalent to 2.5 mg API using the D-P apparatus. Sporanox^®^ OS and Sporanox^®^ Capsules are represented by black circles and grey triangles, respectively. Left: Percentage release profile in the donor chamber. Right: Percentage release profile in the acceptor chamber. Dotted line indicates transition from gastric sector to intestinal sector. * indicates where UV blackout has occurred in the aqueous phase due to precipitation and /or limitations of quantification. Each data point represents the mean ± SD (*n* = 3).

Sporanox^®^ OS was completely dissolved in the gastric environment, whereas only 11.98% ± 1.86% (*n* = 3, ± SD) of the itraconazole from the Sporanox^®^ capsules was in solution at the end of the experiment in the gastric environment, as shown in Figure 9. After the switch to intestinal conditions, significant turbidity was observed for both formulations, indicating that significant precipitation had occurred. The combination of the UV scattering and low drug concentration in the donor chamber meant that it was not possible to accurately quantify itraconazole concentrations in the donor chamber during the experiment in the intestinal environment. This incomplete dissolution of the capsule formulation in the gastric media followed by precipitation upon change to intestinal conditions was consistent with behaviour observed in other in vitro setups [38]. The early flux values into the acceptor chamber for the Sporanox^®^ OS and capsules were 0.99 ± 0.09 and 0.61 ± 0.14 μg·min^−1^·cm^−2^, respectively, whereas the late flux values were 0.41 ± 0.19 and 0.09 ± 0.01 μg·min^−1^·cm^−2^, respectively (*n* = 3, ± SD). At the end of the experiment, a significantly greater amount of itraconazole was present in the acceptor chamber for the OS vs. the capsules (11.42 ± 2.69 vs. 4.00 ± 1.06 μg/mL, *n* = 3, ± SD).

### 3.4. Limitations of UV Probes

The use of UV fibre optic dip probes to quantify drug in situ in both small-scale setups was useful to increase the throughput of the experiments and reduce the risk of precipitation occurring “offline” when analysing samples. Using the second derivative of the UV spectrum was useful to overcome the scattering of light caused by precipitation of drug or by particles of undissolved formulation. A nylon mesh around the UV probe has been used in the literature to effectively reduce scattering of light due to particles [39]. However, using UV dip probes to quantify drug may still be problematic if there is very rapid precipitation of drug and/or quantification limitations (Figure 5, Figure 6 and Figure 9), if the drug molecule itself poorly absorbs UV light or if an excipient in the formulation absorbs light at similar wavelengths to the drug. In such circumstances, it may be sufficient to rely on the concentration profile in the organic layer/acceptor chamber or it may be necessary to take samples offline to quantify drug concentration in the aqueous/donor chamber by HPLC. An assessment of which approach to take should be decided on a case-by-case basis, depending on the characteristics of the drug and formulation being studied.

### 3.5. PBPK Modelling

The values of parameters taken from the literature or estimated experimentally and used in the PBPK models are summarized for each drug in Table 1, Table 2 and Table 3.

The CSR estimated from the solvent shift experiments were 13.33, 16.93 and 44.97 for dipyridamole, ketoconazole and itraconazole, respectively. All experimental values were greater than the simulator default CSR value of 10. The PRC values (h^−1^) estimated from the biphasic dissolution experiments were 1.08, 2.02, 1.98 and 2.11 for dipyridamole, ketoconazole, Sporanox^®^ OS and Sporanox^®^ capsules, respectively. These values are all lower than the simulator default value of 4 h^−1^. Larger PRC values indicate a faster rate of drug precipitation in the intestine. A very similar PRC was calculated for ketoconazole using either the aqueous concentration profile or the partition rate (2.02 vs. 1.99 h^−1^).

The PRC values determined from the D-P experiments were also inputted into the Simcyp^®^ PBPK simulator using the same physiochemical and CSR parameters as those used for the biphasic dissolution modelling. The PRC values (h^−1^) estimated from the flux experiments were 2.02 and 6.14 for dipyridamole and ketoconazole, respectively. Due to the absence of donor chamber concentration data after transition to intestinal conditions and the lag time associated with the flux of drug across the biomimetic membrane, it was not possible to accurately estimate a PRC for the Sporanox formulations using the D-P data.

**Figure 10:**
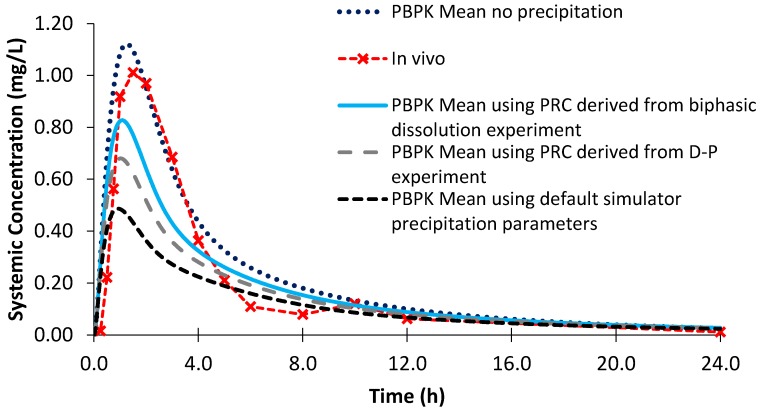
Simulated plasma profiles vs. in vivo plasma profile (dashed line with crosses) of dipyridamole after a single oral dose of dipyridamole 75 mg, based on the design of the clinical study by Ricevuti et al. [22]. The in vivo plasma profile is represented by the red dashed line with crosses. The simulated plasma profile derived from the biphasic dissolution data, the D-P data, the PBPK default simulator parameters and the profile without precipitation are represented by the solid blue line, the dashed grey line, the dashed black line and dotted dark blue line, respectively.

**Figure 11:**
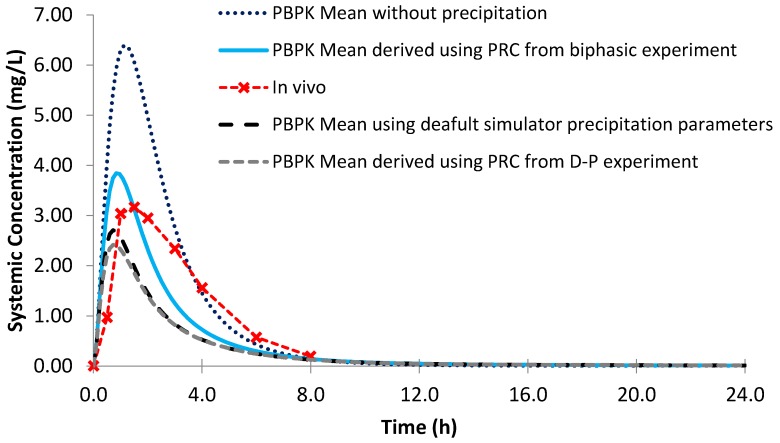
Simulated plasma profile vs. in vivo plasma profile (dashed line with crosses) of ketoconazole after a single oral dose of ketoconazole 200 mg, based on the design of the clinical study by Daneshmend et al. [23]. The in vivo plasma profile is represented by the red dashed line with crosses. The simulated plasma profile derived from the biphasic dissolution data, the D-P data, the PBPK default simulator parameters and the profile without precipitation are represented by the solid blue line, the dashed grey line, the dashed black line and dotted dark blue line, respectively.

The AUC and C_max_ obtained from the PBPK modelling using biphasic dissolution data for both dipyridamole and ketoconazole were within a 25% percentage error (PE) when compared to the in vivo values (Table 4 and Table 5; Figure 10 and Figure 11). The AUC and C_max_ values reported were determined using the individual plasma profiles as opposed to taking values from the mean plasma profiles. Modelling using the D-P data resulted in a larger PE when modelling the AUC and C_max_ of dipyridamole and ketoconazole compared to the biphasic dissolution data. Using simulator default values for PRC and CSR led to a significant underestimation and a greater PE of both AUC and C_max_ in each case relative to the in vivo profiles (Table 4 and Table 5). The effect of precipitation was particularly observed by the impact on C_max_ values, as depending on the dose and solubility of the specific API the impact on AUC may or may not be substantial. These differences in the modelled profiles using the default precipitation parameters or modelling without precipitation highlighted the sensitivity of the models to precipitation.

Modelling the profiles without precipitation led to an overestimation of mean AUC and C_max_ values for both dipyridamole and ketoconazole, leading to a larger PE compared to modelling using the biphasic experimental data (Table 4 and Table 5). However, the effect of modelling without precipitation was greater on the modelled plasma profile of ketoconazole, as dipyridamole has a lower rate of precipitation in the model (1.08 vs. 2.02 h^−1^ using the biphasic experimental information). The PE of the C_max_ for the modelled plasma without precipitation for dipyridamole (Figure 10) was < 25%. As luminal precipitation data corresponding to the dipyridamole formulation tested in the Ricevuti et al. study [22] was not available, it was not possible to directly compare precipitation levels in the upper small intestine between the model and in vivo. A study using an oral solution of dipyridamole found minimal levels of precipitation (≤7%) in the duodenum [40]. However, data after an oral solution may underestimate precipitation compared to the dosage form administered in the Ricevuti et al. study; if dissolution in the stomach is not complete, solid particles could enhance precipitation in the duodenum. Overall, the modelled results indicate that the experimentally derived precipitation parameters had a low or minimal effect on the plasma profile of dipyridamole, whilst modelling using the default precipitation parameters resulted in a large overestimation of precipitation. The effect of precipitation on ketoconazole was more evident from the modelled plasma profiles, with the model without precipitation showing large overestimations in the plasma profile relative to in vivo (Figure 11). This was generally in line with a previous study which found precipitation in the duodenum (≤16%) after administration of a ketoconazole oral solution [40]. As with dipyridamole, modelling using default precipitation parameters resulted in a large overestimation of ketoconazole precipitation.

**Figure 12:**
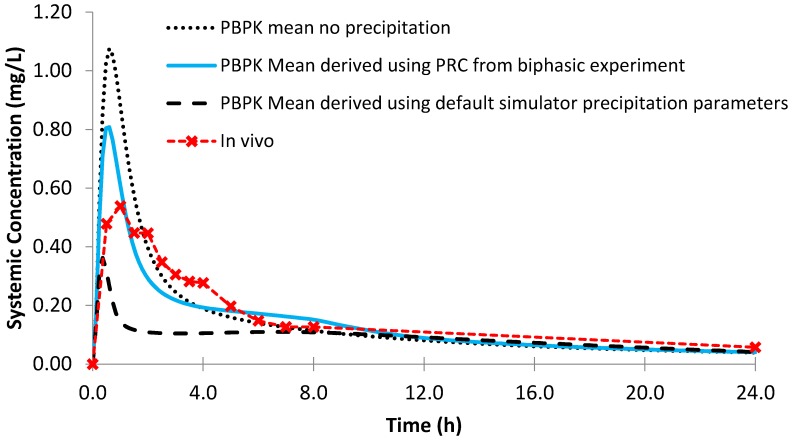
Simulated plasma profile vs. in vivo plasma profile (dashed line with crosses) of itraconazole solution after a single oral dose of itraconazole 200 mg, based on the design of the clinical study by Brouwers et al. [24]. The in vivo plasma profile is represented by the red dashed line with crosses. The simulated plasma profile derived from the biphasic dissolution data, the PBPK default simulator parameters and the profile without precipitation are represented by the solid blue line, the dashed black line and dotted dark blue line, respectively.

Using the biphasic PRC, the modelled AUC for Sporanox^®^ OS was within a 25% PE when compared to the observed in vivo clinical values (Figure 12; Table 6). However, the modelled C_max_ was not adequately captured, as it was 1.56 times greater compared to in vivo [24]. Removing precipitation from the model resulted in an even larger C_max_ value with a greater PE than modelling using the biphasic experimental information. This is in line with the in vivo study, which found substantial intestinal precipitation in the duodenal aspirates after administration of the OS [24]. However, the increase in AUC without precipitation was modest (<7%), suggesting that precipitation had a minor influence on the total exposure of the itraconazole from the OS (Table 6). Using the default precipitation parameters led to an overestimation of precipitation and an underestimation of both AUC and C_max_ values compared to the average plasma profile in adults (Figure 12).

**Figure 13:**
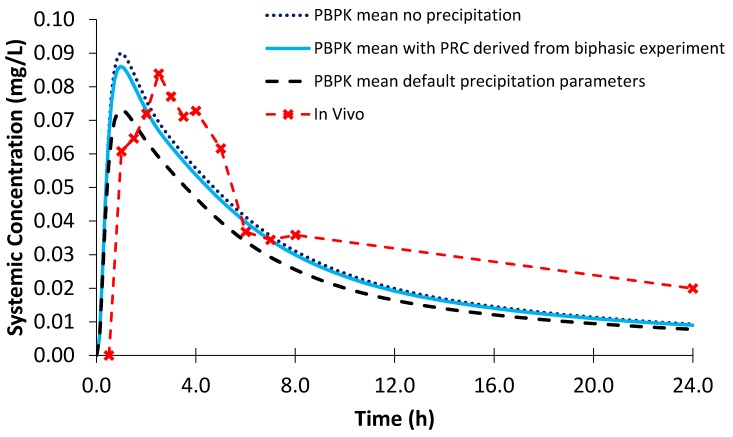
Simulated plasma profile vs. in vivo plasma profile (dashed line with crosses) of itraconazole solid dosage form after a single oral dose of itraconazole 200 mg, based on the design of the clinical study by Brouwers et al. [24]. The in vivo plasma profile is represented by the red dashed line with crosses. The simulated plasma profile derived from the biphasic dissolution data, the PBPK default simulator parameters and the profile without precipitation are represented by the solid blue line, the dashed black line and dotted dark blue line, respectively.

Using the biphasic PRC, the modelled AUC and C_max_ values for Sporanox^®^ capsules were within a 25% PE when compared to the observed in vivo clinical values (Figure 13; Table 7). Modelling without precipitation had a minor effect on the modelled C_max_ and AUC values, suggesting that absorption after administration of Sporanox^®^ capsules is predominantly limited by the solubility of itraconazole in the lumen of the small intestine. Indeed, large amounts of solid itraconazole from the ASD were found in the gastric and duodenal aspirates in the in vivo study [24]. Using the default precipitation parameters, the C_max_ value was adequately estimated (PE < 25%) and AUC underestimated.

Using PBPK modelling facilitates a greater understanding of the factors affecting the oral bioavailability of a molecule. As dipyridamole, ketoconazole and itraconazole are all weakly basic drugs, it could be expected that the pharmacokinetics of each would be highly dependent on the in vivo precipitation in the GI tract. Precipitation of a weakly basic drug in the intestinal lumen can have a significant impact on the bioavailability of a drug [41], potentially affecting the clinical performance of the administered drug. While the modelled profile of ketoconazole was highly dependent on the precipitation of the drug in the intestinal lumen, the modelled profile of itraconazole OS and of dipyridamole were less sensitive to precipitation. Furthermore, the ASD formulation of itraconazole, displayed little sensitivity to precipitation in the PBPK model as it was limited by solubility and dissolution of drug. This greater understanding of the factors affecting the modelled plasma profiles can contribute to model-informed drug development.

It is worth mentioning that simulated profiles constructed using the in vitro data collected in the present study may be improved if a biorelevant estimation of the CSC is performed. In this study, the estimation of CSC was made by injecting a stock solution of the drug in DMSO into the Level II FaSSIF V2. Despite preparing a highly concentrated stock solution to minimise the volume of DMSO added into the test, the presence of DMSO will lead to an overestimation of the CSC. In addition, the in vitro conditions for generating information about the CSC differs greatly to the in vivo environment. In vivo gastric contents usually are not mixed at once with intestinal contents, whereas ‘seed’ particles (undissolved from the stomach) may trigger precipitation in the small intestine. Biorelevant gastrointestinal transfer models have been proposed, but predominantly on a large-scale basis (e.g., the BioGIT and Kostewicz systems) [42,43].

The nature of the sudden transfer from gastric to intestinal conditions was distinct from the more gradual in vivo gastric emptying process or the first order PBPK gastric emptying process [44,45]. As a result, greater precipitation rates are likely to be observed in vitro with the sudden shift in intestinal conditions. In addition, the CSC was likely to be overestimated due to the use of solvent in the experimental setup. However, overestimation of both the CSC and PRC are likely to somewhat confound each other; a higher CSC will mean that precipitation will start later in the model, but this precipitation will occur at a faster rate due to the larger PRC value. The effect of the combination of these errors could lead to a similar AUC compared to in vivo, albeit with a slightly different shape of the plasma profile.

While *T*_max_ was earlier in the models than in vivo, this was reasonable considering the variability observed in the PK profiles of each of the compounds. In later stages of the drug development process, the models could be optimised to improve the fit relative to each of the plasma profiles. However, the purpose of these models was to show the integration of small-scale in vitro experimental results into early stage modelling, especially focusing on precipitation parameters. Therefore, the models were not optimised to achieve a better fit to in vivo data.

Modelling the plasma profiles of the itraconazole formulations involved greater complexity compared to dipyridamole and ketoconazole. The very low solubility of itraconazole means that in vitro analysis can be affected by the limitation of quantification, resulting in less data being available to build the model. In addition, adequately capturing the bio-enabling nature of the itraconazole formulations was challenging. In the case of the OS, the mean maximum cyclodextrin concentration in the duodenum in the PBPK model (18.87 mg/mL) was close to the reported in vivo value (20.5 mg/mL) [46] The cyclodextrin binding was represented by a 1:2 model (1 API molecule to 2 cyclodextrin molecules) as a 1:3 binding model is not yet available in the software. However, considering the concentration range of cyclodextrin in the GI tract, the assessment of the difference in the solubility of itraconazole using the 1:2 or 1:3 binding model was minor.

## 4. Concluding Remarks

After confirming that mixing of the organic layer with Level II FaSSIF components has minimal effect, if any, this study showed that biphasic experiments are useful for estimating PRC values of dipyridamole and ketoconazole. Based on the comparison of the resulted simulated plasma profiles with the actual plasma profiles in adults, estimations were better than those from the D-P data or the default values of the Simcyp software, which have previously been employed to model drug precipitation [47]. Biphasic experiments were also useful for estimating PRC values of itraconazole; for Sporanox^®^ OS, subsequent plasma simulations were better than using default PRC values in the Simcyp software. The biphasic experimental setup seems to be a useful approach for screening APIs with weak base characteristics and for studying enabling drug formulations in early formulation development stages when only small amounts of the APIs are available. While a more mechanistic precipitation model would be useful to develop, it can be difficult to adequately parameterise such a model. This empirical first order model was a simple and pragmatic approach to model precipitation at an early stage.

D-P experiments resulted in an overestimation of luminal precipitation, believed to be related to the slow flux of the drug across the biomimetic membrane into the acceptor chamber. This slow ‘absorption’ of drug in the D-P experiments affected the dynamic dissolution process in the donor chamber as sink conditions were not rapidly regenerated, resulting in a greater precipitation rate. The smaller surface area of the biomimetic membrane (1.54 cm^2^) compared to the interface between the layers in the biphasic experiment (19.63 cm^2^) was a limitation of this setup. Small surface area is a significant issue for D-P systems to overcome when aiming to replicate the rapid intestinal absorption process.

The two small-scale setups evaluated in this study provide biorelevant information which can be incorporated into PBPK modelling at an early stage of development using only small quantities of drug/formulations. These methods are particularly useful to efficiently screen different prospective formulations and gain an insight into their behaviour. At later stages of the development process, formulations can be tested using a full-scale in vitro method, which can provide a better representation of the gastrointestinal transit and luminal conditions, such as the BioGIT [42] or the so-called transfer model [43]. Information obtained from full-scale in vitro testing could then be included in the PBPK model to improve the modelling output [30,48]. In this way, the small-scale biphasic dissolution testing and the full-scale in vitro testing can be viewed as complementary in vitro tools used at different points of the developmental process.

## Figures and Tables

**Table 1:** Values of physicochemical and pharmacokinetic parameters used in physiologically based pharmacokinetic (PBPK) models for dipyridamole in the present study.

Parameter (Units)	Values Used	Refs/Comments
**Physchem and Blood Binding Parameters**		
Mol wt (g/mol)	504.6	
Log Po:w	3.97	In house experimental database
Compound type	Monoprotic base	In house experimental database
pKa	6.07	In house experimental database
Fraction unbound in plasma	0.002	Pathak et al. [25]
Blood plasma ratio	0.56	Predicted in Simcyp
Fraction unbound in enterocyte	1	Simcyp compound file
**Drug Absorption Parameters (ADAM Model)**		
Apparent Caco-2 cell permeability (×10^−6^ cm/s) apical pH 7.4 - basolateral pH 7.4	11.24	Skolnik et al. [26]
Calibrator compounds Caco-2 cell permeability (×10^−6^ cm/s)	Cimetidine 1.64 Propranolol 21.29 Verapamil 22.68 Metoprolol 17.74	Skolnik et al. [26]
Predicted_Peff,man_ (×10^−4^ cm/s)	2.90	Predicted in Simcyp using Caco-2 cell permeability
Aqueous (Aq) intrinsic solubility (mg/mL)	0.0065	Calculated used pH solubility profile
Solubility Factor (SF)	1081	Estimated in Simcyp
Diffusion Layer Model (DLM) scalar	1	Simcyp default value
Particle density (g/mL)	1.2	Default Simcyp value
Particle size distribution	Monodispersed	Default Simcyp value
Particle radius (µm)	10	Default Simcyp value
Log bile micellar: buffer partition coefficient (Log K_m:w_) neutral	4.65	Estimated in Simcyp in vitro analysis (SIVA) toolkit
Log K_m:w_ ion	4.07	Estimated in SIVA
Particle diffusion layer thickness (h_eff_) prediction	Hintz–Johnson method	Default Simcyp method [27]
Critical Supersaturation Ratio (CSR)	13.33	Calculated from experimental data, this study
Precipitation Rate Constant (PRC) (1/h)	1.08 (from biphasic exp)/2.02 (from D-P exp)	Calculated from experimental data, this study
Secondary PRC (sPRC) (1/h)	N/A	
Monomer diffusion coeff (10^−^^4^ cm^2^/min)	3.70	Predicted in Simcyp
Micelle diffusion coeff (10^−^^4^ cm^2^/min)	0.78	Default Simcyp value
**Distribution Parameters**		
Model	Full PBPK Model	
Method	Method 3	
Tissue-plasma partition coefficient (Kp) scalar	1	Pathak et al. [25]
Steady State Volume of Distribution (Vss) (L/kg)	0.41	Predicted within Simcyp
**Elimination Parameters**		
Intravenous clearance (CL_iv)_ (L/h)	12	Persantin^®^ Ampoules 10 mg/2 mL solution for infusion product information [19]
Renal clearance (L/h)	0	Nielsen-Kudsk and Pedersen [28]

**Table 2:** Values of physicochemical and pharmacokinetic parameters used in PBPK models for ketoconazole in the present study.

Parameter (Units)	Values Used	Refs/Comments
**Physchem and Blood Binding Parameters**		
Mol wt (g/mol)	531.4	
Log Po:w	3.84	In house experimental database
Compound type	Diprotic base	In house experimental database
pKa	3.16, 6.13	In house experimental database
Fraction unbound in plasma	0.029	Martinez-Jorda et al. [29]
Blood plasma ratio	0.62	Simcyp inhibitor compound file
Fraction unbound in enterocyte	0.06	Simcyp inhibitor compound file
**Drug Absorption Parameters (ADAM Model)**		
Apparent Caco-2 cell permeability (×10^−6^ cm/s) apical pH 7.4 - basolateral pH 7.4	15.95	Skolnik et al. [26]
Calibrator compounds Caco-2 cell permeability (×10^−6^ cm/s)	Cimetidine 1.64 Propranolol 21.29 Verapamil 22.68 Metoprolol 17.74	Skolnik et al. [26]
Predicted_Peff,man_ (×10^−4^ cm/s)	3.70	Predicted in Simcyp using Caco-2 cell permeability
Aq intrinsic solubility (mg/mL)	0.0064	Back calculated using pH solubility data
Solubility factor	2167	Estimated in SIVA
DLM scalar	1	Simcyp default value
Particle density (g/mL)	1.2	Simcyp default value
Particle size distribution	Monodispersed	Default Simcyp
Particle radius (µm)	12	Pathak et al. [30]
Log K_m:w_ neutral	4.30	Estimated in SIVA
Log K_m:w_ ion	4.28	Estimated in SIVA
Particle h_eff_ prediction	Hintz–Johnson method	Default Simcyp method [27]
CSR	16.93	Calculated from experimental data, this study
PRC (1/h)	2.02 (from biphasic exp)/6.14 (from D-P exp)	Calculated from experimental data, this study
sPRC (1/h)	N/A	
Monomer diffusion coeff (10^−^^4^ cm^2^/min)	3.62	Predicted in Simcyp
Micelle diffusion coeff (10^−^^4^ cm^2^/min)	0.78	Default Simcyp Value
**Distribution Parameters**		
Model	Full PBPK Model	
Method	Method 2	
Kp scalar	0.012	Cristofoletti et al. [31]
Vss (L/kg)	0.2	Predicted within Simcyp
**Elimination Parameters**		
CL_iv_ (L/h)	14.4	Huang et al. [20]
Renal clearance (L/h)	0.15	Pathak et al. [30]

**Table 3:** Values of physicochemical and pharmacokinetic parameters used in PBPK models for itraconazole in the present study.

Parameter (Units)	Values Used	Refs/Comments
**Physchem and Blood Binding Parameters**		
Mol wt (g/mol)	704.64	
Log Po:w	5.66	FDA Label Sporanox
Compound type	Monoprotic base	In house experimental database
pKa	3.87	In house experimental database
Fraction unbound in plasma	0.002	FDA Label Sporanox
Blood plasma ratio	0.58	Simcyp inhibitor compound file
Fraction unbound in enterocyte	0.016	Simcyp inhibitor compound file
**Drug Absorption Parameters (ADAM Model)**		
MCDK II (×10^−6^ cm/s)	57.1	Simcyp inhibitor compound file
Calibrator compounds MCDK II permeability (×10^−6^ cm/s)	Cimetidine 2.00 Propranolol 49.60 Verapamil 59.10	Simcyp inhibitor compound file
Predicted_Peff,man_ (×10^−4^ cm/s)	9.85	Predicted in Simcyp using MCDK cell permeability
Aq intrinsic solubility (mg/mL)	8.94E-05	Back calculated using pH solubility data
Solubility factor	222.37	Estimated in SIVA
DLM scalar	1	Simcyp default value
Particle density (g/mL)	1.2	Simcyp default value
Particle size distribution	Monodispersed	Simcyp default value
Particle radius (µm)	10	Simcyp default value
Log K_m:w_ neutral	5.62	Predicted in SIVA
Log K_m:w_ ion	5.48	Predicted in SIVA
Particle h_eff_ prediction	Hintz–Johnson method	Default Simcyp method [27]
CSR	44.97	Calculated from experimental data, this study
PRC (1/h)	1.98 (OS), 2.11 (capsules)	Calculated from experimental data, this study
sPRC (1/h)	N/A	
Monomer diffusion coeff (10^−4^ cm^2^/s)	3.17	Predicted within Simcyp
Micelle diffusion coeff (10^−^^4^ cm^2^/s)	0.78	Default Simcyp Value
**Solid state 2 (Capsules only)**		
Aq. intrinsic solubility (mg/ml)	0.001	Matsui et al. [32]
DLM scalar	0.69	Estimated in SIVA from dissolution profile
**Excipient Mediated Solubility (OS only)**		
Binding constant (M^−1^)	1654 (K1:1), 13 (K1:2)	Peeters et al. [33]
Binding constant (M^−1^) stomach	9895 (K1:1), 23 (K1:2)	Peeters et al. [33]
**Distribution Parameters**		
Model	Full PBPK Model	
Method	Method 2	
Kp scalar	0.19	Poirier et al. [34]
Vss (L/kg)	11.06	Predicted within Simcyp
**Elimination Parameters**		
CL_iv_ (L/h)	22.9	Heykants et al. [21]
Renal clearance (L/h)	0	Simcyp inhibitor compound file

**Table 4:** Observed and modelled drug exposure pharmacokinetic parameters for 75 mg immediate release tablets of dipyridamole.

PK Parameter	Ricevuti et al. ± SD	PBPK Using Experimental Biphasic InForm PRC Value ± SD (% PE)	PBPK Using Experimental D-P PRC Value ± SD (% PE)	PBPK Using Default Simulator Precipitation Values ± SD (% PE)	PBPK with no Precipitation ± SD (% PE)
AUC (mg/L h)	4.13 ± 0.52	4.12 ± 1.49 (0.24%)	3.58 ± 1.32 (13.32%)	2.81 ± 1.13 (31.96%)	5.28 ± 1.98 (27.85%)
C_max_ (mg/L)	0.93 ± 0.13	0.83 ± 0.21 (10.75%)	0.71 ± 0.18 (23.66%)	0.49 ± 0.15 (47.31%)	1.14 ± 0.29 (22.58%)

**Table 5:** Observed and modelled drug exposure pharmacokinetic parameters for 200 mg immediate release tablets of ketoconazole.

PK Parameter.	Daneshmend et al. ± SD	PBPK Using Biphasic Experimental Biphasic InForm PRC Value ± SD (% PE)	PBPK Using Experimental D-P PRC Value ± SD (% PE)	PBPK Default Simulator Precipitation Values ± SD (% PE)	PBPK with no Precipitation ± SD (% PE)
AUC (mg/L h)	12.9 ± 1.50	10.49 ± 4.37 (18.68%)	7.08 ± 3.27 (45.12%)	7.44 ± 3.41 (42.33%)	17.93 ± 7.87 (38.99%)
C_max_ (mg/L)	4.36 ± 0.54	4.01 ± 1.23 (8.03%)	2.52 ± 0.99 (42.20%)	2.80 ± 1.00 (35.78%)	6.53 ± 2.06 (49.77%)

**Table 6:** Observed and modelled drug exposure pharmacokinetic parameters for 200 mg dose of Sporanox oral solution.

PK Parameter	Brouwers et al. ± SD	PBPK Using Experimental Biphasic InForm PRC and CSR Values ± SD (% PE)	PBPK Using Experimental D-P PRC Value ± SD (% PE)	PBPK Using Default Simulator Precipitation Values ± SD (% PE)	PBPK with no Precipitation ± SD (% PE)
AUC (mg/L h)	3.64	3.32 ± 1.12 (8.79%)	D-P PRC value not available *	2.18 ± 0.78 (40.11%)	3.52 ± 1.17 (3.30 %)
C_max_ (mg/L)	0.54	0.84 ± 0.23 (55.56%)		0.37 ± 0.14 (31.48%)	1.10 ± 0.28 (103.70%)

* Due to the UV black-out and the slow flux into the donor chamber, it was not possible to estimate the precipitation rate constant (PRC) for both Sporanox formulations using data from the D-P experiments.

**Table 7:** Observed and predicted drug exposure pharmacokinetic parameters for 200 mg dose of Sporanox capsules.

PK Parameter	Brouwers et al. ± SD	PBPK Using Experimental Biphasic InForm PRC Value ± SD (% PE)	PBPK Using Experimental D-P PRC Value ± SD (% PE)	PBPK Using Default Simulator Precipitation Values ± SD (% PE)	PBPK with No Precipitation ± SD (% PE)
AUC (mg/L h)	0.87	0.67 ± 0.35 (22.99%)	D-P PRC value not available *	0.58 ± 0.31 (33.33%)	0.70 ± 0.36 (19.54%)
C_max_ (mg/L)	0.084	0.089 ± 0.045 (5.95%)		0.076 ± 0.037 (9.52%)	0.092 ± 0.047 (9.52%)

* Due to the UV black-out and the slow flux into the donor chamber, it was not possible to estimate the PRC for both Sporanox formulations using data from the D-P experiments.

## Supplementary Materials

The following are available online at https://www.mdpi.com/1999-4923/12/3/272/s1. Table S1: Detection Wavelengths used to quantify drugs with the fibre optic UV dip probes, Figure S1: Modelling first order PRC using the natural log vs. time profile for ketoconazole using the aqueous phase date from biphasic dissolution experiment, Figure S2: Modelling PRC using the partition rate into decanol from the biphasic experiment.
