# Supplementary material for: On the Usefulness of Two Small-Scale In Vitro Setups in the Evaluation of Luminal Precipitation of Lipophilic Weak Bases in Early Formulation Development
Source: Pharmaceutics. 2020 Mar 16;12(3):272. doi: 10.3390/pharmaceutics12030272 (PMC7151110; doi:10.3390/pharmaceutics12030272)
Supplement: Supplementary file 1 [file pharmaceutics-12-00272-s001.pdf]

Supplementary Materials: On the Usefulness of Two Small-Scale In Vitro Setups in the Evaluation of Luminal Precipitation of Lipophilic Weak Bases in Early Formulation Development

Table S1. Detection Wavelengths used to quantify drugs with the fibre optic UV dip probes.

API	Fibre Optic Detection Spectral Range (nm)		
	**Ionised (pH 2)**	**Unionised (pH 6.8)**	**In Decanol**
**Dipyridamole**	400–450	400–460	400–450
**Ketoconazole**	260–280	280–300 (direct)/255–265, 288–298 (2nd derivative)	285–315
**Itraconazole**	260–300	275–290	250–300

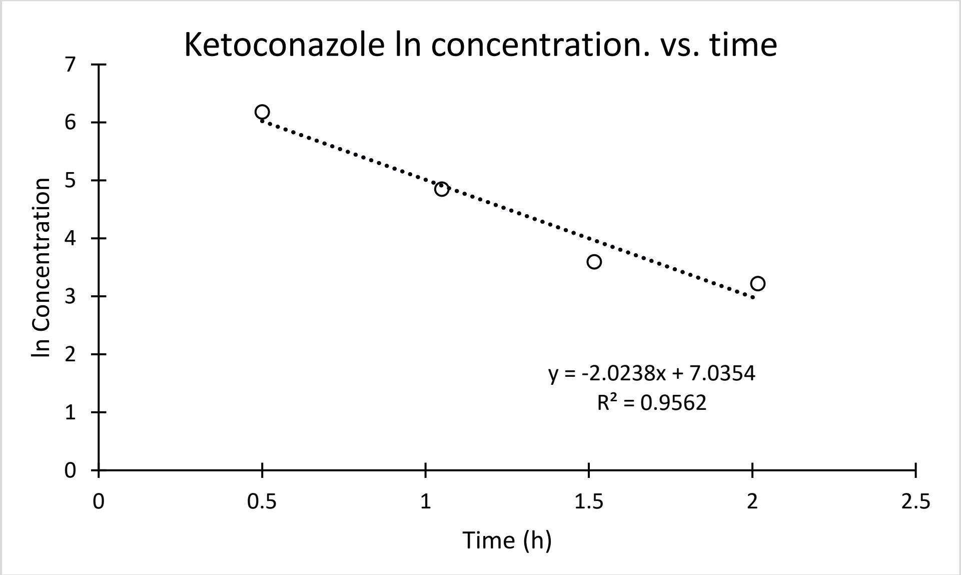

Figure S1. Modelling first order PRC using the natural log vs. time profile for ketoconazole using the aqueous phase date from biphasic dissolution experiment.

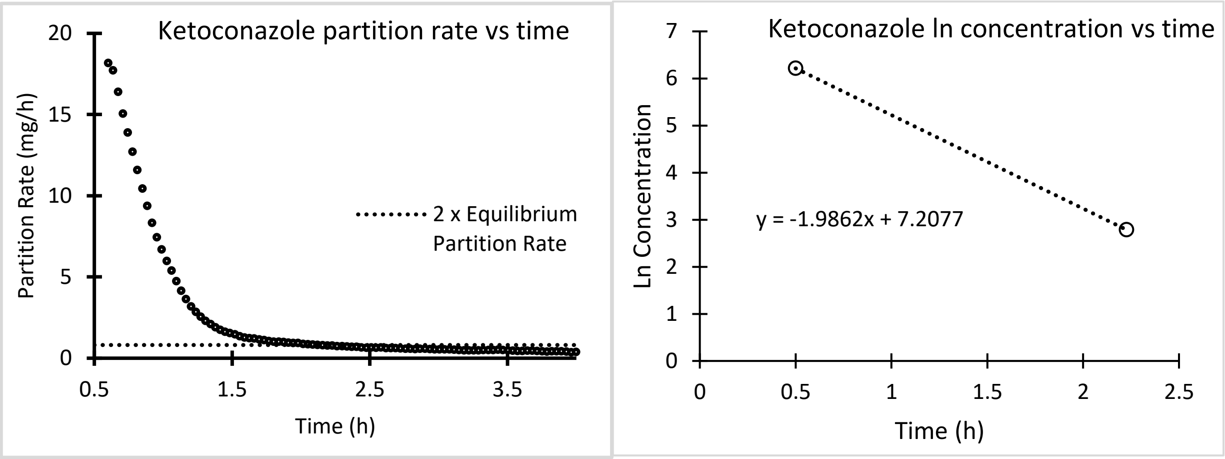

Figure S2. Modelling PRC using the partition rate into decanol from the biphasic experiment. The time to reach equilibrium solubility was estimated using the change in partition rate of drug into the decanol layer; this was determined as the first time point at which the partition rate fell below double the equilibrium partition rate of the compound into the decanol layer. A first order precipitation rate was calculated using the initial concentration in the aqueous phase upon transition to intestinal conditions and the estimated time to reach equilibrium solubility in the intestinal sector. Left ketoconazole partition rate into decanol vs time, dotted line showing double the equilibrium partition rate. Right natural log concentration of the aqueous phase vs. time profile.
